# Supplementary material for: Senescent cell turnover slows with age providing an explanation for the Gompertz law
Source: Nat Commun. 2019 Dec 2;10:5495. doi: 10.1038/s41467-019-13192-4 (PMC6889273; doi:10.1038/s41467-019-13192-4)
Supplement: Supplementary file 1 — Supplementary Information [file 41467_2019_13192_MOESM1_ESM.pdf]

Supplementary Information for

Senescent cell turnover slows with age providing an explanation for the Gompertz law

Karin et al.

Supplementary Note 1. Stochastic modeling of longitudinal trajectories of SnCs in mice.

Model comparison and best-fit parameters.

In this section, we consider different stochastic models for the dynamics of senescent cell (SnC) abundance, denoted by X. X is removed at rate $removal$ and produced at rate $production$, and includes a noise term *noise*:

$$\dot{X} = production - removal + noise$$

We modeled four biological processes: (i) SnC production rate can increase with age due accumulation of mutations, telomere damage and other forms of intracellular damage that can trigger cellular senescence (*1*), (ii) SnCs can catalyze their own production by paracrine effects (*2*), (iii) SnC removal can decrease with age due to age-related decline in immune surveillance function (*3*) and (iv) SnCs can slow their own removal, for example by saturating or downregulating their own immune surveillance mechanisms. All combinations of these options (including or discluding i-iv) lead to 2^4=16 different circuits:

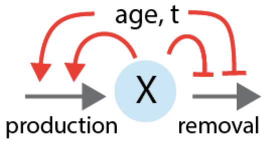

The model that includes all four processes is

$$\dot{X} = (\eta_0 + \eta_1 t)(1 + \eta_2 X) - \frac{\beta_0 - \beta_1 t}{1 + \beta_2 X} X + \sqrt{2\epsilon}\xi_t$$

Where X is SnC abundance, t is time, η_0 is the initial SnC production rate, η_1 is the increase in SnC production rate with age, η_2 is the autocatalysis rate, β_0 is the initial removal rate, β_1 is the decrease in removal rate with age, β_2^{-1} is the half-way saturation point for removal and ϵ is the noise amplitude. The most general model has, therefore, 7 parameters.

We defined variants of this model, by all combinations of setting model parameters to zero. A model with no age-related rise in production has η_1=0, a model with no autocatalysis has η_2=0, a model with no age-related decline in removal has β_1=0, and a model with no saturation of removal has β_2=0. The scanned models also include models where there is no removal (i.e. $\beta_0 = \beta_1 = 0$). In this way, we scanned all 1-parameter models, 2-parameter models, etc., up to the full 7-parameter model.

For each model, we scanned parameters to find the parameters that maximize the log-likelihood of the measured longitudinal SnC trajectories (as described in the methods section). We performed the parameter scan for each model in several steps. First a sparse grid was used, in which each parameter was assigned values spaced uniformly on a log scale between e^{-10} to e^{1}. Each parameter could also be assigned 0, and in total the number of parameter sets in each grid was at least 50,000 parameter sets. We then used a finer and finer uniform grid within the 95% bootstrapped confidence intervals estimated by the previous sparser scan, until convergence was reached. The scans yielded the following estimates for the log-likelihood of each model.

Model #	Model	#Params (best fit)	Saturating removal	Log-likelihood	BIC
1	$\dot{X} = (\eta_0 + \eta_1 t) - \beta_0 X + \sqrt{2\epsilon}\xi_t$	4 (4)	no	-535	1092
2	$\dot{X} = (\eta_0 + \eta_1 t)(1 + \eta_2 X) - \beta_0 X + \sqrt{2\epsilon}\xi_t$	5 (5)	no	-507	1042
3	$\boldsymbol{\dot{X} = \eta_0 - (\beta_0 - \beta_1 t)X + \sqrt{2\epsilon}\xi_t (USR\ model)}$	**4 (3)**	**no**	**-500**	**1023**
4	$\dot{X} = (\eta_0 + \eta_1 t) - (\beta_0 - \beta_1 t)X + \sqrt{2\epsilon}\xi_t$	5 (3)	no	-500	1028
5	$\dot{X} = \eta_0(1 + \eta_2 X) - (\beta_0 - \beta_1 t)X + \sqrt{2\epsilon}\xi_t$	5 (3)	no	-500	1028
6	$\dot{X} = (\eta_0 + \eta_1 t)(1 + \eta_2 X) - (\beta_0 - \beta_1 t)X + \sqrt{2\epsilon}\xi_t$	6 (3)	no	-500	1034
7	$\dot{X} = \eta_0 - \frac{\beta_0 - \beta_1 t}{1 + X\beta_2}X + \sqrt{2\epsilon}\xi_t$	5 (5)	yes	-483	994
8	$\dot{X} = \eta_0(1 + \eta_2 X) - \frac{\beta_0 - \beta_1 t}{1 + X\beta_2}X + \sqrt{2\epsilon}\xi_t$	6 (6)	yes	-479	992
9	$\boldsymbol{\dot{X} = (\eta_0 + \eta_1 t) - \frac{\beta_0}{1 + X\beta_2}X + \sqrt{2\epsilon}\xi_t\ (SR\ model)}$	**5 (4)**	**yes**	**-475**	**978**
10	$\dot{X} = (\eta_0 + \eta_1 t) - \frac{\beta_0 - \beta_1 t}{1 + X\beta_2}X + \sqrt{2\epsilon}\xi_t$	6 (4)	yes	-475	984
11	$\dot{X} = (\eta_0 + \eta_1 t)(1 + \eta_2 X) - \frac{\beta_0}{1 + X\beta_2}X + \sqrt{2\epsilon}\xi_t$	6 (6)	yes	-473.7	982
12	$\dot{X} = (\eta_0 + \eta_1 t)(1 + \eta_2 X) - \frac{\beta_0 - \beta_1 t}{1 + X\beta_2}X + \sqrt{2\epsilon}\xi_t$	7 (6)	yes	-473.7	987

Supplementary Table 1. Maximum log-likelihood scores for models. Number of parameters in parenthesis the effective number of parameters needed for maximum likelihood, since in some models some of the best-fit parameters are negligible

Models without time-dependence of parameters, that is, with $\eta_1 = \beta_1 = 0$, show very low likelihood (LL<-540) and are not shown. Capping log-likelihood of sub-trajectories with very low-likelihood at LL=-10 or LL=-15, to prevent sensitivity to outliers, yields similar results.

As can be seen from the table above, the best-fit models all have saturating removal ($\beta_2 > 0$). The best-fit model shows removal rates of days in young mice and weeks in old mice. The USR model, which is the best-fit model with unsaturated removal ($\beta_2 = 0$), has a poorer likelihood, and show much longer half-life for SnC. The minimal best-fit model is the 4-parameter SR model with linearly increasing production and constant maximal removal rate, which we rewrite as:

$$\dot{X} = \eta t - \frac{\beta X}{\kappa + X} + \sqrt{2\epsilon}\xi_t$$

(where $\beta = \frac{\beta_0}{\beta_2}, \kappa = \beta_2^{-1}$). Adding temporal changes in the parameters κ, ϵ does not yield improved fits.

The best-fit parameters for the SR model are shown in Supplementary Table 2, with confidence intervals obtained by bootstrapping using the mice individuals in the dataset. SnC levels are given in arbitrary units [au] such that young mice have a mean level of 1[au] in the luciferase dataset.

Parameter	Mean	SE	5%CI	95%CI
$\boldsymbol{\eta}$	$4.2 \cdot 10^{-4}[au]day^{-2}$	$0.5 \cdot 10^{-4}$	$3.5 \cdot 10^{-4}$	$5 \cdot 10^{-4}$
$\boldsymbol{\beta}$	$0.27\ day^{-1}$	0.04	0.21	0.32
$\boldsymbol{\kappa}$	$1.1\ [au]$	0.3	0.7	1.8
$\boldsymbol{\epsilon}$	$0.14[au]^2day^{-1}$	0.02	0.1	0.18

Supplementary Table 2. Best fit parameters for SR model.

These parameters provide a good fit for the mean ($\frac{\chi^2}{N} = 1.27, N = 10$), autocorrelation ($\frac{\chi^2}{N} = 0.63$), and skewness ($\frac{\chi^2}{N} = 0.79$) of the data. The fit for the standard deviation has a higher error ($\frac{\chi^2}{N} = 3.3$), due to the extremely low standard error of the standard deviation at week 16, which is a clear outlier compared with week 8 and week 24. When excluding this point (week 16), we get a good fit for the standard deviation as well ($\frac{\chi^2}{N} = 1, N = 9$), as well an improved fit for the mean ($\frac{\chi^2}{N} = 0.94$) and skewness ($\frac{\chi^2}{N} = 0.37$), and a similar fit for autocorrelation ($\frac{\chi^2}{N} = 0.67$).

The next best-fit minimal model, without saturation, is the 3-parameter model highlighted in Supplementary Table 1. This model is called unsaturated removal (USR model). The best fit parameters for this model are the following: $\beta_0 = 0.07 \pm 0.018\ day^{-1}$, $\beta_1 = 0.044 \pm 0.01\ day^{-1}year^{-1}$, $\epsilon = 0.22 \pm 0.03\ [au]^2day^{-1}$. This model, with its best-fit parameters, describes the data poorly (orange lines in Fig 2C, $\frac{\chi^2}{N} = 7.18$ or $\frac{\chi^2}{N} = 6.15$ without week 16 for mean SnC). These parameters are also the best fit parameters for all models without saturation where $\beta_1 > 0$. Here is the full list of best-fit parameters (selected from the simulation that provided the maximum likelihood):

Model #	$\boldsymbol{\eta_0}$ $[au]day^{-2}$	$\boldsymbol{\eta_1}[au]day^{-1}$	$\boldsymbol{\eta_2}$ $[au]^{-1}$	$\boldsymbol{\beta_0}$ $[day]^{-1}$	$\boldsymbol{\beta_1}$ $[day]^{-2}$	$\boldsymbol{\beta_2}$ $[au]^{-1}$	$\boldsymbol{\epsilon}$ $[au]^2day^{-1}$
**1**	0.004	0.0001	0	0.019	0	0	0.1
**2**	0.004	0.00006	0.6	0.03	0	0	0.14
**3**	0	0	0	0.07	0.00012	0	0.22
**4**	0	0	0	0.07	0.00012	0	0.22
**5**	0	0	0	0.07	0.00012	0	0.22
**6**	0	0	0	0.07	0.00012	0	0.22
**7**	0.15	0	0	0.8	0.0009	1.9	0.19
**8**	0.15	0	0.04	0.9	0.0009	2.5	0.11

**9**	0	0.000425	0	0.25	0	0.9	0.1425
**10**	0	0.000425	0	0.25	0	0.9	0.1425
**11**	0.35	0.00055	0.04	0.9	0	0.9	0.2
**12**	0.35	0.00055	0.04	0.9	0	0.9	0.2

Supplementary Table 3. Best fit parameters for models specified in Supplementary Table 1.

The best fit parameters without time-dependence of parameters (portrayed in Figure 2) are $\eta_0 = 1.075[au]day^{-2}, \beta_0 = 0.75\ day^{-1}, \epsilon = 0.11[au]^2 day^{-1}$.

Robustness of parameter estimation to experimental noise.

We next tested whether the estimate of the best-fit parameters is robust to experimental noise. We modeled experimental noise as log-normally-distributed multiplicative noise with mean 1 and standard deviation σ. We repeated the procedure to calculate the log-likelihood of the data (Methods), except that we multiplied the starting point and ending point of each simulated sub-trajectory by a random number sampled from the noise distribution. We then found the best-fit parameters and confidence intervals as described above. The maximal log-likelihood for the SR model, when considering experimental noise with $\sigma = 0.2$ (20% noise), is improved compared to no noise (-465 compared with -475), and we find the following best-fit parameters:

Parameter	Mean	SE	5% CI	95% CI
$\boldsymbol{\eta}$	$4 \cdot 10^{-4}[AU]day^{-2}$	$0.7 \cdot 10^{-4}$	$2.4 \cdot 10^{-4}$	$6 \cdot 10^{-4}$
$\boldsymbol{\beta}$	$0.28\ day^{-1}$	0.055	0.17	0.47
$\boldsymbol{\kappa}$	$1.6[AU]$	0.4	0.6	2.7
$\boldsymbol{\epsilon}$	$0.13[AU]^2 day^{-1}$	0.02	0.1	0.18

Supplementary Table 4. Best fit parameters for SR model, assuming 20% noise.

The resulting best-fit parameters are similar to the best-fit parameters without considering experimental noise, and lead to the same conclusions regarding SnC dynamics, turnover rate, saturation and slowdown. Similar results are found with noise amplitudes of $\sigma = 0.05, 0.1, 0.3$. Therefore, the present parameters are robust to experimental noise in the longitudinal SnC measurements.

Best-fit parameters adjusted for mortality.

We sought to find the best-fit parameters for the SR model $\dot{X} = \eta t - \frac{\beta X}{\kappa + X} + \sqrt{2\epsilon}\xi_t$, that describe both the longitudinal trajectories and mice mortality statistics. For this purpose, we scanned the values of β and κ and constrained η, ϵ so the mean and standard deviation of the mortality-time distribution will be within 2% of the data for the mortality distribution of WT (C57BL/6J) mice obtained from the Mouse Phenome Database (*4*) (see Methods). We set mortality as described in the Methods section.

In a simple realization of the connection between SnC and mortality, one can assume that death occurs when SnC level exceed X_C. We set $X_C = 17[au]$, which is the maximal observed SnC level in the normalized TBL data. The best-fit maximum likelihood parameters are:

Parameter	Mean	SE	5% CI	95% CI
$\boldsymbol{\eta}$	$2.3 \cdot 10^{-4}[au]day^{-2}$	$0.25 \cdot 10^{-4}$	$1.9 \cdot 10^{-4}$	$2.9 \cdot 10^{-4}$
$\boldsymbol{\beta}$	$0.15\ day^{-1}$	0.022	0.12	0.2
$\boldsymbol{\kappa}$	$0.5[au]$	0.1	0.4	0.7
$\boldsymbol{\epsilon}$	$0.16[au]^2 day^{-1}$	0.02	0.14	0.2

Supplementary Table 5. Best fit parameters for SR model, adjusted for mortality.

The mortality distribution computed with these best-fit parameters agrees with the mouse mortality distribution (p>0.2 under both Kolmogorov-Smirnov and Anderson-Darling tests). The log-likelihood for the best-fit parameters for the longitudinal SnC data is -490. When excluding the outlier point (week 16), these parameters provide a good fit for the mean ($\frac{\chi^2}{N} = 0.84$, N=9), skewness ($\frac{\chi^2}{N} = 0.28$), and autocorrelation ($\frac{\chi^2}{N} = 0.46$), though the fit for the variance is worse than the parameters of Supplementary Table 2 ($\frac{\chi^2}{N} = 2.9$).

While these best-fit parameters lead to similar conclusions regarding SnC turnover rate, saturation and slowdown, they are different from the best-fit parameters that are not constrained by mortality (Supplementary Table 2). Specifically, while noise amplitude ϵ is similar to the original best-fit value, the other parameters are smaller, leading to higher noise compared with feedback strength. The reason for this may be slightly different survival curves for mice grown in different locales, and/or that the requirement that death occurs at a critical SnC level may be unrealistic compared with a more gradual dependence of mortality on SnC. Such gradual dependence allows for lower noise compared with feedback (Supplementary Note 2).

Mathematical modelling of the bystander effect of SnC production

Several recent studies suggest that SnC can be produced as a result of direct induction by neighboring SnCs ("bystander effect") as a result of paracrine interactions (*5–8*). The injection of a small number of senescent cells can spread cellular senescence in host tissues (*9*), providing *in vivo* evidence for the bystander effect.

The contribution of the bystander effect to SnC dynamics can be modelled in several ways. The first and most straightforward is that SnC production rate includes a term that is proportional to SnC abundance $\eta_2 X$. This assumes that each SnC generates more SnCs at a rate η_2, leading to exponential propagation of SnCs in the tissue. As can be seen in Supplementary Table 1, this mechanism is not essential to explain the data of Burd et al, because models that include such a bystander effect term do not outperform models without the bystander effect. In the most complex model: $\dot{X} = (\eta_0 + \eta_1 t)(1 + \eta_2 X) - \frac{\beta_0}{1 + X\beta_2^{-1}} X + \sqrt{2\epsilon}\xi_t$, the contribution of the bystander effect η_2 is negligible in the best fit parameter set.

Another possibility is that the bystander effect is non-linear. For example, it may be that SnCs only catalyze their own formation when they are at a high concentration. This nonlinearity can create a local tipping point, where SnCs are induced at a rate higher than their removal once they exceed a certain concentration.

Lastly, it may be that the bystander effect is limited to a specific bounded region within a tissue, or that the paracrine induction of SnCs weakens after near neighbors turn senescent. Future work can address this by considering the spatial dynamics of SnCs in tissues.

Models with individual variation in parameters

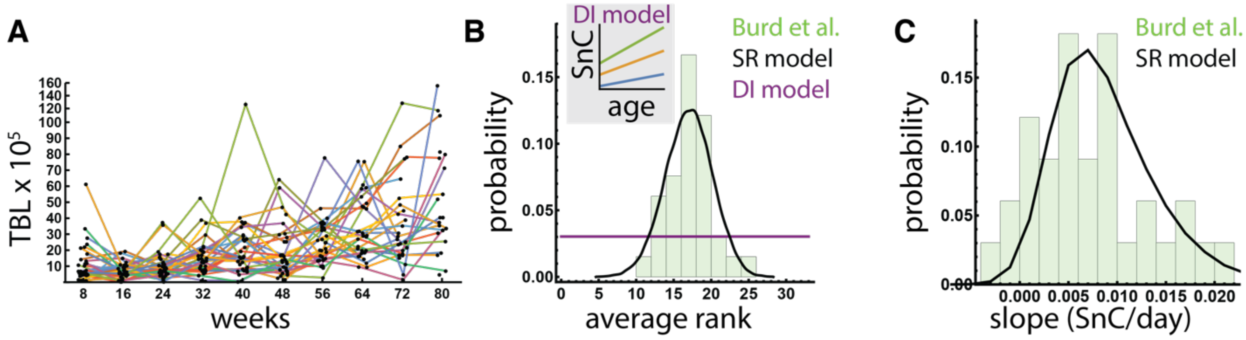

Supplementary Figure 1. Individual variation in SnC accumulation rate can be described by stochastic models. (A) Individual trajectories of SnC accumulation from Burd et al., as in Figure 1A. Each color denotes a different mouse. (B) Mean rank of every individual from Burd et al. (light green) compared with rank distributions from a model of Deterministic Individuality (DI, purple, model illustrated in inset) or the SR model (black). (C) Slope of SnC accumulation rate for every individual from Burd et al. (light green) compared with slope distribution predicted from the parametrized SR model.

So far, we modeled individual variation in SnC accumulation as resulting from stochasticity in X which we described using a white noise term $\sqrt{2\epsilon}\xi_t$. Variation in senescent cell accumulation may also result from inter-individual variation in model parameters. This inter-individual variation in

parameters is relevant for the case of humans, who vary in genotype and environment. There is also likely to be some variation in model parameters between the inbred mice, whose SnC levels were measured longitudinally by Burd et al (Supplementary Figure 1A). To test whether it is justified to model variation in model parameters between individuals, we estimated the persistent variation between individuals. For this, we calculated the rank of every individual at each time point, and estimated the mean rank of every individual throughout their lifetime (Supplementary Figure 1B). In the most extreme case of inter-individual variation, where individuals have separate trajectories of SnC accumulation throughout their lifetime, we expect this distribution to be uniform (every individual maintains its rank throughout its lifetime, a 'deterministic individuality' model). On the other hand, the stochastic variation described by the parametrized SR model suggests a much narrower variation in mean rank, which is similar to what is observed in the dataset.

Finally, we estimated the variation in SnC accumulation by calculating the slope of SnC accumulation for every individual (Supplementary Figure 1C). This corresponds well with the variation in SnC accumulation rates described by the parametrized SR model (Supplementary Figure 1C). We conclude that stochastic models are adequate for describing variation in SnC dynamics for the Burd et al. dataset.

We note that the comparison of models to the longitudinal data in the main text was done with the full data trajectories, and not to their average statistics.

Additional models with nonlinear mechanisms.

In the circuit scan described so far, we showed that saturation of the SnC removal mechanism is required to explain the longitudinal trajectories of Burd et al. The saturation effect is modeled using a Michaelis-Menten term for the SnC-dependent reduction in removal rate, $\frac{1}{1+\beta_2 X}$. This term introduces a non-linear dependence on SnC abundance, X. The autocatalysis effect, on the other hand, is modeled using a linear dependence on SnC abundance. To test whether other non-linear effects may explain the longitudinal trajectories, we tested several other plausible models. We kept the removal term linear in X, and added non-linearity to the production term.

First, we tested models where there is no saturation, and the non-linearity is introduced from logistic effects. In the first model, the logistic effect is introduced in the total production of SnC,

$$\dot{X} = (\eta_0 + \eta_1 t)(1 + \eta_2 X)(1 - \eta_3 X) - (\beta_0 - \beta_1 t)X + \sqrt{2\epsilon}\xi_t$$

and in the second model, it is introduced specifically in the autocatalysis factor:

$$\dot{X} = (\eta_0 + \eta_1 t)(1 + \eta_2 X(1 - \eta_3 X)) - (\beta_0 - \beta_1 t)X + \sqrt{2\epsilon}\xi_t$$

Both models do not improve on the USR model (same equations with $\eta_3 = 0$). They have a log-likelihood of -500, and the best-fit values for η_0, η_1 are 0.

We also tested a model where autocatalysis has a quadratic term $\eta_3 X^2$:

$$\dot{X} = (\eta_0 + \eta_1 t)(1 + \eta_2 X + \eta_3 X^2) - (\beta_0 - \beta_1 t)X + \sqrt{2\epsilon}\xi_t$$

This model improves on the other USR models, and has a maximal log-likelihood of $L = -486$ (BIC=1012). This model is much worse than the best-fit SR model (ΔBIC=34). We therefore conclude that these models are insufficient to explain the longitudinal trajectories.

Longitudinal trajectories of mice carrying a transgenic allele of human p16 fused to luciferase.

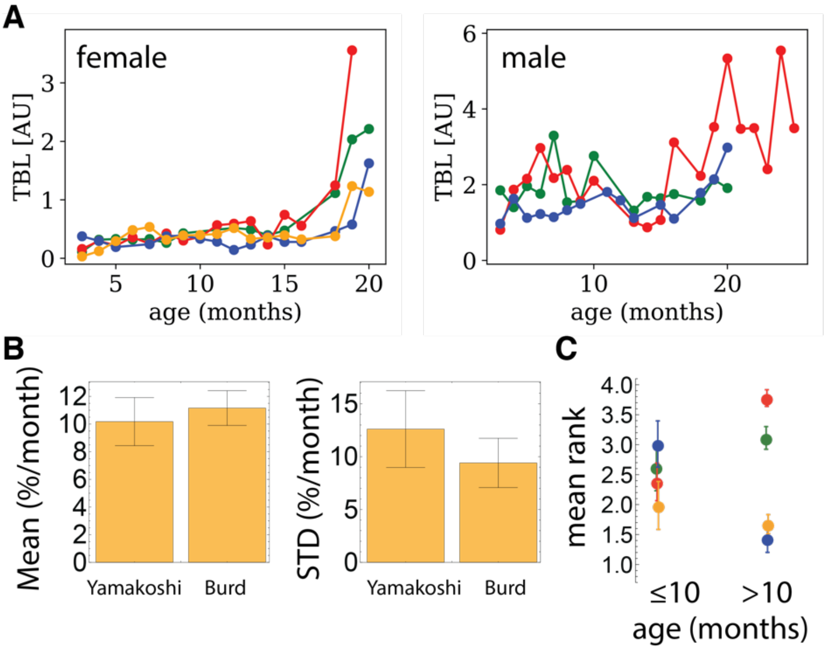

Supplementary Figure 2. Longitudinal mouse luciferase trajectories from a human p16 construct in the presence of native p16 loci. (A) Total body luminescence (TBL) in transgenic mice with the human p16 construct tagged with luciferase from Yamakoshi et al. (*10*). Each curve represents an individual mouse. (B) For the female mice in the Yamakoshi et al. dataset, both the mean and the standard deviation increase at a similar rate to that of the Burd et al. dataset. (C) Mean TBL rank of female mice in the Yamakoshi datasets, before/after the age 10 months. The trajectories of the female mice in the Yamakoshi dataset show higher persistence at old ages (individuals with high TBL stay high). Source data are provided as a Source Data file.

Quantitative modelling of the dynamics of SnCs during ageing requires longitudinal measurements of SnCs in individual mice *in vivo*. In the main text, we analyzed such a dataset collected by Burd et al. (*11*), which included luciferase-based measurements of SnC abundance in 33 individual mice, from early age (8 weeks) to middle-late adulthood (80 weeks). The measurements were based on a knock-in allele, in which the firefly luciferase gene was targeted into one of the endogenous p16 loci. While the luciferase output retains the cis-regulatory elements of p16, the resulting mouse is heterozygous for p16. We therefore tested longitudinal measurements of p16 based on another method.

For this, we obtained data from Yamakoshi et al. (*10*), which created a transgenic mouse model with a human p16 gene tagged with luciferase, maintaining the native p16 loci. The dataset contains 7 mice (3 male and 4 female), whose total body luminescence was monitored every month (Supplementary Figure 2A). Luciferase output increased with age for these mice. Unlike in the Burd et al. dataset, there were marked differences in $p16^{LUC}$ between male and female mice in this dataset. While young females in this dataset had very low luciferase output (as in the Burd dataset), young males had luciferase output that was already similar to that of old females. The dataset for the males also had

many missing values, with a third of the time-points containing only a single mouse. For this reason, we chose not to aggregate the measurements and to focus our analysis on the female mice.

The Yamakoshi dataset is much smaller than the dataset of Burd et al. (4 mice compared with 33 mice), and we therefore cannot fit it with a dynamical model. However, it resembles the Burd et al. dataset in several important quantitative aspects. The mean luciferase measurements increase at a similar rate with age to the Burd et al. dataset (10% ± 2% per month compared with 10% ± 1%), as does the standard deviation (12% ± 3% per month compared with 9% ± 1%) (Supplementary Figure 2B). In addition, as predicted by the SR model, the Yamakoshi et al. mice show more persistent variation at old ages than at young ages. This can be quantified by the mean rank, which mixes rapidly in the young (<=10mo) showing a mean rank around 2-3, but stays more persistent in the old (>10mo): the individuals with high SnC remain high and those with low SnC remain low, Supplementary Figure 2C. These similarities indicate that the Burd et al and Yamkoshi et al constructs may report similar underlying dynamics.

Supplementary Note 2. Analytical properties of the SR model

In this section, we derive various analytical properties of the SR model.

The SR model equation is:

$$\dot{X} = \eta t - \frac{\beta X}{X+\kappa} + \sqrt{2\epsilon}\xi_t \qquad [1]$$

One can write this using a potential U(X,t):

$$\dot{X} = -\frac{d}{dx}U(X,t) + \sqrt{2\epsilon}\xi_t \qquad [2]$$

Where the potential is

$$U(X) = (\beta - \eta t)X - \beta\kappa \log(\kappa + X) \qquad [3]$$

since:

$$-\frac{d}{dx}U(X,t) = -(\beta - \eta t) + \frac{\beta\kappa}{X+\kappa} = \eta t + \frac{-\beta(\kappa + X) + \beta\kappa}{X+\kappa} = \eta t - \frac{\beta X}{X+\kappa}$$

Quasi-stationary distribution and statistical properties of SR model

Due to the rapid turnover relative to the organism lifetime, we can use a quasi-steady-state approximation (main text Figure 1B). At quasi-steady state:

$$X_{ST} = \frac{\eta\kappa t}{\beta - \eta t} [4]$$

In a quasi-steady state approximation, the stationary distribution is given by the Boltzmann distribution:

$$Prob[X = x] \propto e^{-\frac{U(x)}{\epsilon}} \qquad [5]$$

Therefore:

$$Prob[X = x] \propto e^{-\frac{(\beta-\eta t)x}{\epsilon}}(\kappa + x)^{\frac{\beta\kappa}{\epsilon}} \qquad [6]$$

With the normalization factor, the distribution is:

$$Prob[X = x] = \frac{e^{-\frac{x(-\eta t+\beta)}{\epsilon} - \frac{(-\eta t+\beta)\kappa}{\epsilon}}\kappa^{-1-\frac{\beta\kappa}{\epsilon}}\left(-\frac{(\eta-\beta)\kappa}{\epsilon}\right)^{\frac{\epsilon+\beta\kappa}{\epsilon}}(x+\kappa)^{\frac{\beta\kappa}{\epsilon}}}{\text{Gamma}[1 + \frac{\beta\kappa}{\epsilon}, -\frac{(\eta t-\beta)\kappa}{\epsilon}]} [7]$$

We can use this distribution to approximate the mean SnC abundance:

$$\langle X \rangle = \int_{x=0}^{\infty} x\, Prob[X = x]dx = \frac{\kappa\eta t + \epsilon + \frac{e^{\frac{\kappa(\eta t-\beta)}{\epsilon}}\epsilon}{\text{ExpIntegralE}[-\frac{\kappa\beta}{\epsilon}, \frac{\kappa(-\eta t+\beta)}{\epsilon}]}}{\beta - \eta t} [8]$$

Where ExpIntegralE described the exponential integral function $\text{ExpIntegralE}[n, z] = \int_1^{\infty} \frac{e^{-zt}}{t^n} dt$.

For parameters in the relevant range: $\frac{e^{\frac{\kappa(\eta t-\beta)}{\epsilon}}\epsilon}{\text{ExpIntegralE}[-\frac{\kappa\beta}{\epsilon}, \frac{\kappa(-\eta t+\beta)}{\epsilon}]} \ll \kappa\eta t + \epsilon$ this yields

$$\langle X \rangle \approx \frac{\kappa\eta t + \epsilon}{\beta - \eta t} [9]$$

For example, for parameters similar to the best fit mouse parameters, $\beta = 0.2, k = 1, \epsilon = 0.5$, the analytical solution of Supplementary Equation 8 and its approximation Supplementary Equation 9 are nearly identical, as shown in Supplementary Figure 3.

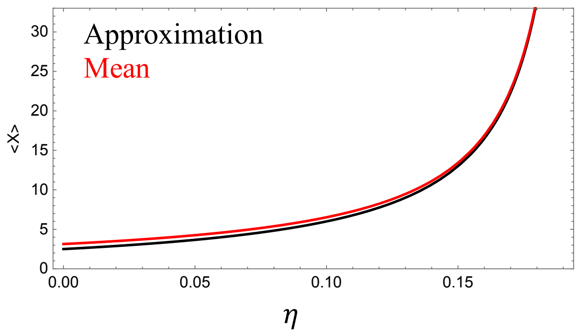

Supplementary Figure 3. Analytical approximation of Supplementary Equation 9 is similar to Supplementary Equation 8 over the relevant range for η. Equations 8,9 were simulated given the physiologically relevant parameters $\beta = 0.2, k = 1, \epsilon = 0.5$, and varying η (corresponding to the dynamics of aging under the SR model). Both equations predict similar mean SnC level <X>.

The variance is estimated as:

$$\langle X^2 \rangle = \frac{\epsilon\left(\kappa\beta + \epsilon - \frac{e^{\frac{\kappa(\eta t-\beta)}{\epsilon}}\left(e^{\frac{\kappa(\eta t-\beta)}{\epsilon}}\epsilon + kp\,\mathrm{ExpIntegralE}\left[-\frac{\kappa\beta}{\epsilon}, \frac{\kappa(-\eta t+\beta)}{\epsilon}\right]\right)}{\mathrm{ExpIntegralE}\left[-\frac{\kappa\beta}{\epsilon}, \frac{k(-\eta t+\beta)}{\epsilon}\right]^2}\right)}{(\eta - \beta)^2} \approx \frac{\kappa\beta + \epsilon^2}{(\eta t - \beta)^2} \qquad [10]$$

The distribution of SnC in the SR model is skewed to the right (Supplementary Equation 7) quantitatively matching the skewness observed in the mouse data (main text Figure 2EF).

Derivation of Gompertz mortality in the SR model

We model mortality as the first time when $X > X_C$ (we later discuss other cases). Thus, death time is a first-passage time of the SR model variable X. To estimate the hazard rate (probability of death per unit time), we apply the Kramer approximation for the first passage time (*12*, *13*):

$$h \approx \frac{\sqrt{U''(X_{ST})U''(X_C)}}{2\pi} e^{-\frac{U(X_C)-U(X_{ST})}{\epsilon}}$$

Where the effective potential U is given by Supplementary Equation 3. For the Gompertz law(*14–18*) to hold, one needs $\frac{U(X_C)-U(X_{ST})}{\epsilon}$ to decrease linearly with time, so that $h \approx e^{\alpha t}$.

The exponent of the hazard rate in the SR model indeed shows the required linearity in time:

$$-\frac{U(X_C) - U(X_{ST})}{\epsilon} = \frac{(\kappa + X_C)\eta t - X_C\beta + \kappa\beta \cdot \mathrm{Log}\left[\frac{(\kappa+X_C)(\beta-\eta t)}{\kappa\beta}\right]}{\epsilon} \qquad [8]$$

The curvature around steady-state is $U''(X_{ST}) = \frac{(\beta-\eta)^2}{\kappa\beta}$. We also denote $U''(X_C) = \omega_{max}$ as the (unknown) curvature around the critical threshold. We thus find that:

$$h \approx \frac{\sqrt{\omega_{max}}}{2\pi}(\kappa + X_C)^{\frac{\kappa\beta}{\epsilon}}(\kappa\beta)^{-\frac{\kappa\beta}{\epsilon}-0.5}(\beta - \eta t)^{\frac{\kappa\beta}{\epsilon}+1} e^{\frac{(\kappa+X_C)\eta t - X_C\beta}{\epsilon}} \qquad [9]$$

The hazard rises exponentially with time as $e^{\alpha t}$ with $\alpha = \frac{(\kappa + X_C)\eta}{\epsilon}$. The model also shows a deceleration in the rise of the hazard rate at very old ages (when $\eta t \approx \beta$), due to the prefactor $(\beta - \eta t)^{\frac{\kappa\beta}{\epsilon}+1}$, as observed for empirical hazard (*19*, *20*). Note that this approximation begins to be inaccurate when $\eta t > \beta$, and simulations of the full SR model are needed to compute the hazard curve at old ages.

Models without SnC turnover (models with $\beta_0 = \beta_1 = 0$) do not yield a mortality distribution that follows the Gompertz law, but, instead, an inverse Gaussian distribution (*21*).

Near-exponential rise of mean SnC in the SR model

The dependence of mean SnC on time in the SR model is similar to an exponential, in the sense that it matches the second order Padé approximation of an exponential:

$$k \cdot e^{\frac{\eta_1}{\beta}t} \approx k \frac{1 + \frac{\eta_1}{\beta}t}{1 - \frac{\eta_1}{\beta}t} = \frac{k\beta + k\eta_1 t}{\beta - \eta_1 t} \approx \frac{\kappa\eta_1 t + \epsilon}{\beta - \eta_1 t} \approx \langle X \rangle \quad [10]$$

The last equality holds when $k\beta \approx \epsilon$. This is the case for the best-fit mouse parameters. More generally, the resemblance to the exponent holds when k, β, ϵ are of similar magnitude (as can be shown by simulation). A semi-logarithmic plot of mean SnC as a function of age in the SR model appears nearly linear (model 9, Supplementary Table 1), whereas the USR with best fit parameters (model 3, Supplementary Table 1) deviates from linearity.

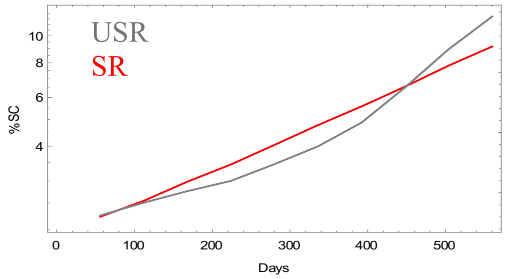

Supplementary Figure 4. Mean SnC increases approximately exponentially for the SR model and super-exponentially for the USR model. The USR model (model 3 in Supplementary Table 1) the SR model (model 9 in Supplementary Table 1) were simulated with their best-fit parameters (Supplementary Table 4). The increase in mean SnC is approximately exponential for the SR model and super-exponential for the USR model. This indicates that the SR model provides a nearly exponential increase in SnC levels as observed.

Gompertz law holds to a good approximation under a general dependence of mortality on SnC level.

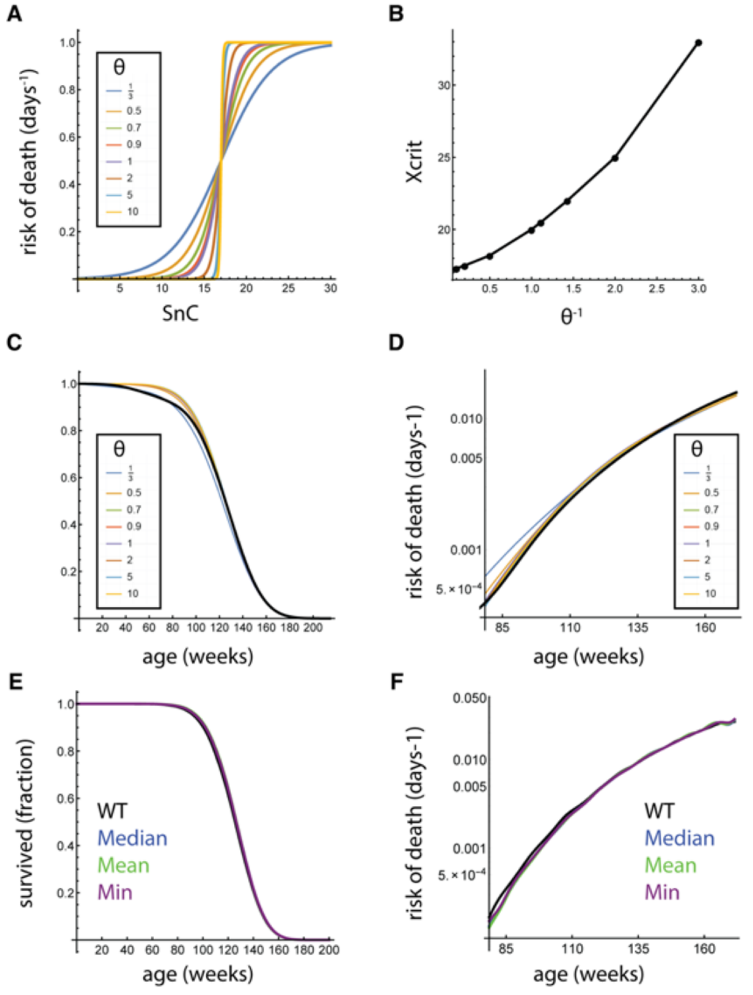

Supplementary Figure 5. Gompertz law holds under a general dependence of mortality on SnC level. (A) We tested whether the Gompertz law holds in the SR model under the more general assumption that probability of death increases with SnC level X as a logistic function with steepness θ and half-way point X_C. In the case of $\theta \gg 1$ this converges to a step function in which death occurs when crossing a threshold X_C. (B) Using the best-fit parameters provided in Section 1, Supplementary Table 5, we show that for each steepness θ we can choose an appropriate X_C such that both the hazard curves (C) and the mortality curves (D) fit the observed mouse mortality statistics well (black curves in panels C-F). (EF) We tested whether the Gompertz law holds under the more general assumption that death occurs only when the average/median/minimal number of SnC over a longer time period (30 days) exceeds a thresholds X_C. We show that with appropriate choices of X_C ($X_C = 17$ for mean, median, $X_C = 15$ for min), both the hazard curves (E) and the mortality curves (F) fit the observed mouse mortality statistics well.

In the previous section we showed that the SR model leads to the Gompertz law, assuming that death occurs when SnC level X crosses a critical level X_C. Here we show by simulation that this conclusion also holds under a more general dependence of death on SnC level. We model probability of death as a function of X as a sigmoidal increasing function with different degrees of steepness. The probability of death is described using a family of logistic functions, in which steepness depends on a parameter θ:

$$Prob(death, x) = \frac{1}{1 + e^{-\theta(X - X_C)}}$$

The larger θ, the steeper the dependence of probability of death on *X,* around the half-way point X_c (Supplementary Figure 5A, similar results are found using Hill functions). For each of the above choices of α one can choose an appropriate X_C so that mortality distributions are very similar to the distribution observed for mice (Supplementary Figure 5B-D), keeping the other model parameters constant at their values in Supplementary Table 5. This indicates that an exponentially rising increase of mortality with age (Gompertz law) plus slowdown at old ages is a property that holds even when assuming a graded probability of death as a function of SnC level.

We also tested whether the Gompertz law holds if we assume that death occurs when SnC exceed a critical threshold for a period of time (30 days). We tested the cases where death occurs when average SnC, median SnC or minimal SnC over 30 days exceed a critical threshold. For each of the above we can choose an appropriate X_C so that mortality distributions are very similar to the distribution observed for mice (Supplementary Figure 5EF), suggesting the generality of Gompertz law for this condition as well.

Supplementary Note 3. Gating strategy employed to identify senescent cells.

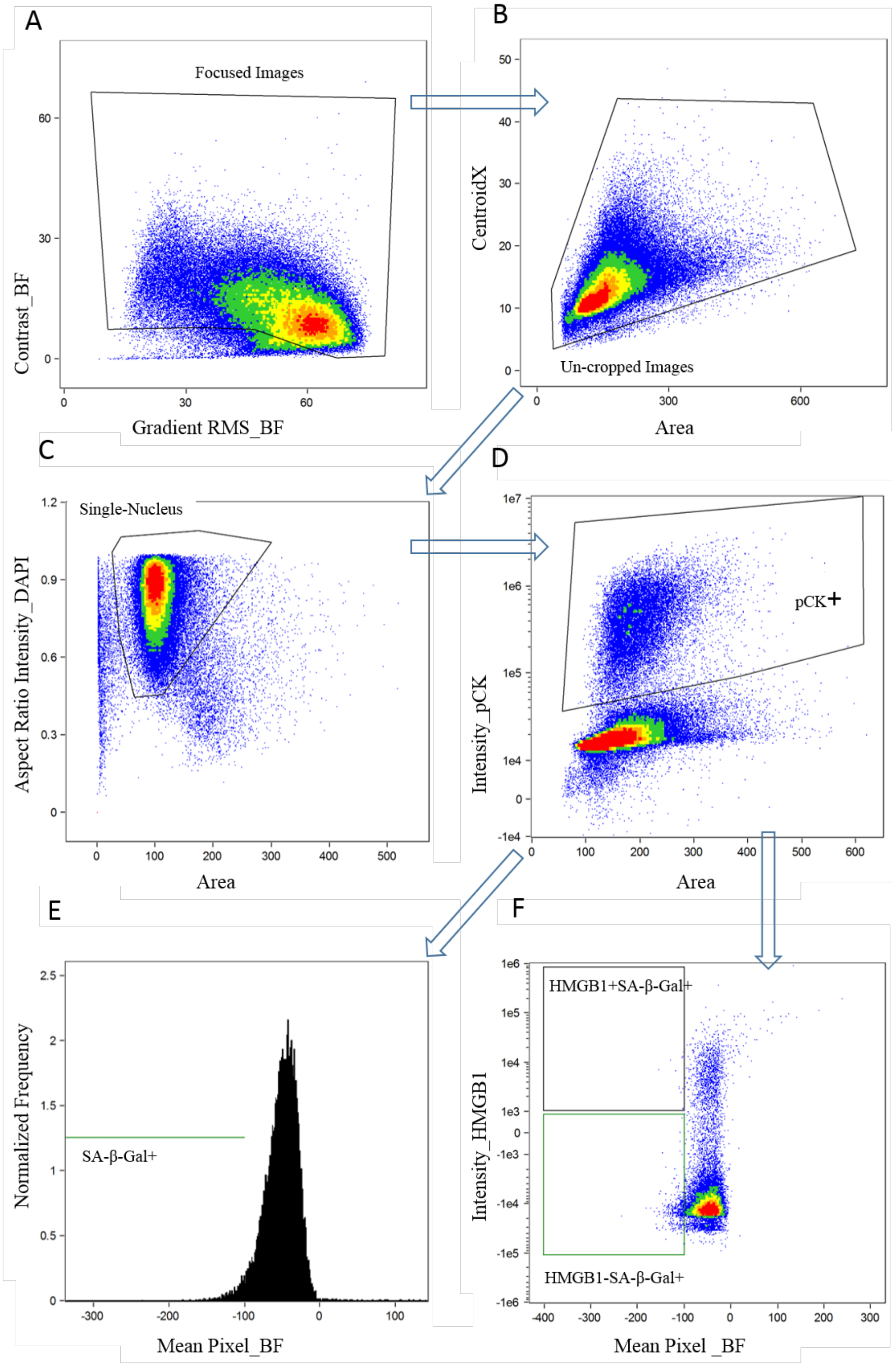

Supplementary Figure 6. Gating strategy employed to identify SA-β-Gal+ or SA-β-GAL+HMGB1- cells in the lung epithelium using ImageStreamX. Gating strategy employed to identify SA-β-Gal+ or SA-β-GAL+HMGB1- cells in the lung epithelium using ImageStreamX. **(A)** Focused images were gated using Gradient Root Mean Square and contrast (both measure the sharpness quality of an image by detecting large changes of pixel values in the image) of the Bright Field (BF) channel. **(B)** Uncropped images were gated out of Focused images using CentroidX (distance of a cell from the left side of the acquired image) and Area in square microns of the bright field image, **(C)** Single nucleus cells were selected using area and aspect ratio (normalized for intensity) of the DAPI staining out of uncropped images, **(D)** pan cytokeratin (pCK) positive cells were selected using intensity and the bright field area out of single nucleus cells, **(E)** Out of pCK positive cells, SA-β-

Gal+ cells were selected using the mean pixel (the mean of the background-subtracted pixels contained in the input mask) of the Bright Field, where SA-β-Gal+ cells are darker and show lower values as previously described (*22*). **(F)** SA-B-Gal+ and HMGB1- cells were gated using HMGB1 intensity and mean pixel of the Bright Field channel.

Supplementary Note 4. Estimation of SnC removal rate.

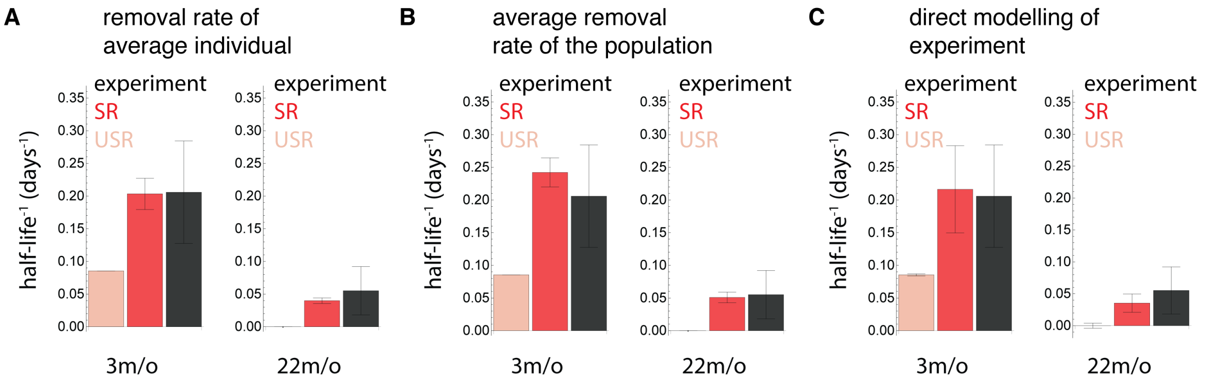

Supplementary Figure 7. Estimation of SnC removal rates by different methods yields similar results. SnC removal rate of the SR production-removal model that was fit to the longitudinal trajectories was estimated by different methods (red bars): (A) Removal rate of the average individual: $\frac{\beta}{\kappa+\bar{X}_i}\log(2)^{-1}$, (B) Average removal rate of the population: $\overline{\frac{\beta}{\kappa+X_i}}\log(2)^{-1}$, and (C) Modeling of bleomycin perturbation experiments. Model predictions were compared with estimation from bleomycin perturbation experiments (Figure 2 in the main text). The model shows similar predictions across estimation methods (red, mean and SE were calculated by bootstrapping according to the number of mice in each bleomycin experiment). Removal rate for the next best-fit model that does not have saturation (USR mode, gray) was estimated in a similar manner.

In this section, we compare three ways to use the SR model to simulate the bleomycin experiment and compute the half-life of SnC levels. We find that the three ways provide very similar results.

The SR production-removal model for SnC dynamics:

$$\dot{X} = \eta t - \frac{\beta X}{\kappa + X} + \sqrt{2\epsilon}\xi_t$$

has a per-SnC removal rate of $\frac{\beta}{\kappa+X}$, where X is the total SnC abundance. Therefore, a perturbation in which SnC abundance is changed by δ ($\delta \ll X$) will decay with a half-life^{-1} of $\frac{\beta}{\kappa+X+\delta}\log(2)^{-1} \approx \frac{\beta}{\kappa+X}\log(2)^{-1}$.

Since SnC abundance X is heterogeneous in the population, the model predicts that SnC removal rate will also be heterogeneous. We considered three ways to estimate the typical removal rate at a given age i.

1. The removal rate of the average individual: $\frac{\beta}{\kappa+\bar{X}_i}\log^{-1} 2$ where $\bar{X}_i$ is the mean SnC level at age i. This calculation predicts a SnC half-life of about 5 ± 1 days in young (3-month-old) mice and a SnC half-life of about 25 ± 6 days in old (22-month-old) mice.
2. The average removal rate of the population: $\overline{\frac{\beta}{\kappa+X_i}}\log^{-1} 2$.This calculation predicts SnC half-life of about 4 ± 1 days in young (3-month-old) mice and a half-life of about 20 ± 5 days in old (22-month-old) mice.

Both estimates yield similar predictions for SnC dynamics that are consistent with the half-life estimated from the perturbation experiments (Figure 2 in the main text, Supplementary Figure 7AB).

3. We also modeled the experiments directly, by bootstrapping SnC levels X_j at age i and simulating the ODE: $\dot{x} = \eta i - \frac{\beta x}{\kappa + X_j}$.We set an initial post-bleomycin level $x\ (5\ days) = X_j + 50[au]$, representing a perturbation that increases lung epithelium SnC level to $50[au]$ after 5 days (the conclusions are not dependent on the exact initial value). For each time point of the bleomycin experiment $T_j \in \{T_1 \dots T_n\}$ we sampled X_j and simulated the above ODE, and measured the level of $x(T_j)$. We then estimated SnC removal rate in the same manner as for the bleomycin time-series (Supplementary Figure 7C, Figure 2C). This method yields an estimated removal rate that is similar to that predicted by methods 1, 2.

We estimated removal time in the same manner for the USR model: $\dot{X} = \eta - (\beta_1 - \beta_0 t)X + \sqrt{2\epsilon}\xi_t$. Since the best-fit USR model predicted a slightly negative removal rate for 22-month-old mice, we set its removal rate at that age to zero. All estimation methods show that the USR model provides a poorer prediction for the SnC half-life (Supplementary Figure 7).

Supplementary Note 5. SR model for SnC dynamics.

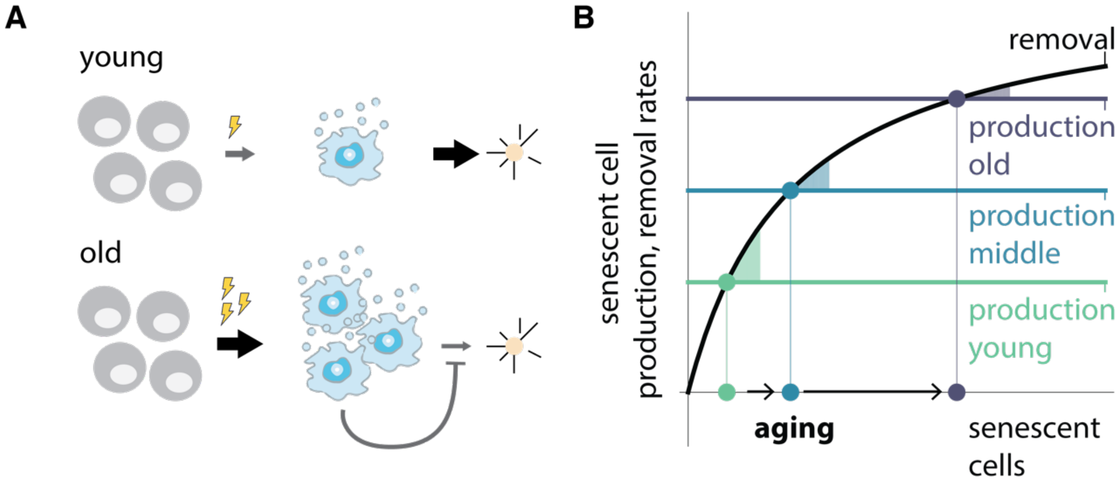

Supplementary Figure 8. Saturating removal and increasing production lead to accelerated SnC abundance with age and persistent SnC fluctuations. (A) With age, production rate increases, SnC levels rise and increasingly saturate their own removal mechanism, further amplifying their rate of increase. (B) Removal and production rates plotted as a function of SnC abundance. Steady-state occurs when production and removal curves cross (dots). As production rises, SnC levels accelerate to higher abundance. At old ages, the spare capacity for removal (the distance between production and removal curves, shaded regions) shrinks, so that SnC perturbations last longer, leading to persistent fluctuations. These fluctuations cause non-genetic variations between individuals that last for long times.

Supplementary Note 6. Predicted effect of senolytic drug regimes

We employed the SR model to simulate the effects of drugs that eliminate SnCs, known as senolytic drugs (*7*, *23–29*). Due to toxicity concerns, it may be desirable to establish regimes of low dose and large temporal spacing for these drugs (*25*). We computed the effect of regiments of senolytic drug treatments as a function of their intake frequency and efficacy.

To model the effect of a senolytic drug, we consider $X_{SENSITIVE}$ to be the number of senescent cells that are sensitive to the drug and $X_{INSENSITIVE}$ to be the number of senescent cells that are not sensitive to the drug ($X_{INSENSITIVE}$ may also represent other forms of damage that impacts aging besides senescent cells). Overall, the level of X is: $X = X_{SENSITIVE} + X_{INSENSITIVE}$.We assume that a fraction $\zeta$of SnC production and noise goes to make $X_{SENSITIVE}$, and the rest $(1-\zeta)$ goes to $X_{INSENSITIVE}$:

$$\dot{X}_{SENSITIVE} = \zeta\eta t - \frac{\beta X_{SENSITIVE}}{X+\kappa} + \sqrt{2\zeta\epsilon}\xi'_t$$

$$\dot{X}_{INSENSITIVE} = (1-\zeta)\eta t - \frac{\beta X_{INSENSITIVE}}{X+\kappa} + \sqrt{2(1-\zeta)\epsilon}\xi''_t$$

All parameters are the same as for mouse (Supplementary Table 5), and death occurs when $X > X_C = 17[AU]$. To model the effect of a drug given every ϕ days, we define a term $f(\phi, \alpha, t)$ which represents the killing rate of the drug:

$$f(\phi, \alpha, t) = \begin{cases} \alpha & if\ t\ mod\ \phi = 0 \\ 0 & otherwise \end{cases}$$

Thus ϕ is the period at which the drug (time between doses) is taken and α is the drug efficacy (rate of killing sensitive cells). Thus:

$$\dot{X}_{SENSITIVE} = \zeta\eta t - f(\phi,\alpha,t) X_{SENSITIVE} - \frac{\beta X}{X_{SENSITIVE} + \kappa} + \sqrt{2\zeta\epsilon}\xi_t$$

We set ζ in mice by assuming that the pharmacogenetic experiments of Baker et al.(*30*), that used a genetic construct to destroy p16-expressing cells, mimic the effect of a drug with a maximal SnC removal rate of $\alpha_{MAX} = 1 day^{-1}$. In these experiments, $\phi = 1 day$ and mean lifespan of the mice increased by 25%. This allows calibrating ζ, providing $\zeta \approx 0.25$. Simulating the model for different ϕ, α gives[38] the following dependence of predicted life extension on the time between doses and efficacy per dose:

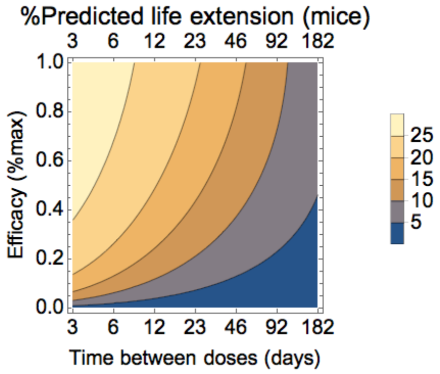

The parameters used for the simulations are the mortality-adjusted best-fit parameters (Supplementary Table 5). We can thus see that effective treatment can be as infrequent as the SnC turnover time (~month in old mice) even for a drug that kills only a fraction of the sensitive SnC.

Supplementary Note 7. Human aging dynamics and the SR model.

To study human mortality curves we used the Swedish life table for 2009, obtained from http://www.lifetable.de. This dataset is standard for demographic research on mortality statistics (*16*, *31–33*). We model the hazard using the hazard from the SR model plus an age-independent extrinsic mortality, $h = h_{SR} + h_{extrinsic}$, with $h_{extrinsic} = 0.4 \cdot 10^{-3} year^{-1}$ estimated from mean mortality at age 20 (Makeham term (*34*)). As discussed in the main text, taking the mortality-adjusted mouse parameters in the SR model (Supplementary Table 5) and adjusting η by ~1/60 is sufficient to obtain a good fit to the human mortality data, without adjusting additional parameters (Figure 3).

To test the plausibility of these parameters, we compared the model predictions to measurements of SnC concentrations in various human tissues using different methods (Supplementary Table 6).

Tissue (Human)	Method	N (number of subjects)	SnC accumulation slope [1/year]
**T-cells (*35*)**	p16 mRNA content	156	3.7±0.4%
**Bone marrow stromal cells (hMSC) (*36*)**	SA-beta-gal	16	3.5±1%
**Renal arteries (*37*)**	%p16-positive nuclei	30	2±1%
**Renal glomureli (*37*)**	%p16-positive nuclei	36	2.1±1.1%
**Renal interstitium (*37*)**	%p16-positive nuclei	35	4±1.5%
**Renal tubules (*37*)**	%p16-positive nuclei	39	4±0.9%
**Corneal epithelium (*38*)**	IHC staining of p16	12	3.5±1.2%
**Cartilage chondrocytes (*39*)**	SA-beta-gal	15	2.0%±0.5%

Supplementary Table 6. SnC accumulation slope for various human tissues.

Data was taken from the figures in the indicated studies. Data points with very small SnC abundance (log value less than -1) were capped at -1. Since we only seek to model SnC levels at adulthood, we used data points after age 20 (results are unaffected by using data at all ages). Slope was estimated using regression on log data, with bootstrapped error bars. The measurements show an approximately exponential increase of SnC levels with a rate of 2%-4% per year. This rate of increase is consistent with the present values for κ and β for mice, which provide an increase of ~2.2% per year in total body SnC. We conclude that κ and β mouse parameters are a plausible choice for human SnC accumulation rates.

Supplementary Note 8. SR model and mortality interventions in *Drosophila*

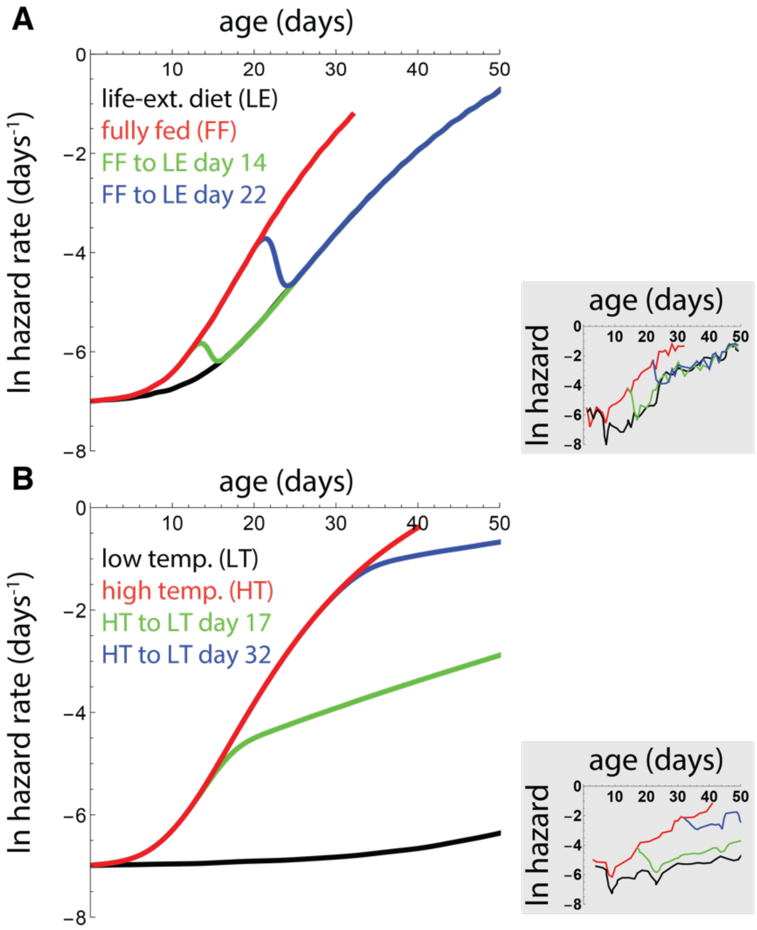

Supplementary Figure 9. Rapid turnover and saturating removal can explain the effect of mid-life interventions on *Drosophila* mortality. (A) The SR model with saturation and rapid turnover of damage ($\beta = 1\ hr^{-1}$, $\kappa = 1[AU]$, $\epsilon = 1\ [AU]^2 hr^{-1}$,$X_C = 15[AU]$), and a slow increase in damage production ($\eta = 0.03[AU]hr^{-1}day^{-1}$) can explain the hazard curve of fully fed *Drosophila* (red curve). A slower rate of increase in damage production $\eta = 0.02[AU]hr^{-1}day^{-1}$ can explain the hazard curve of *Drosophila* under dietary change (LD, black curve). An instantaneous change in η leads to rapid reversal of mortality rates (green and blue lines), explaining the observation of Mair et al. (insets). (B) The SR model with a slow rate of increase in damage production $\eta = 0.005[AU]hr^{-1}day^{-1}$ can explain the hazard curve of *Drosophila* raised at 18C (low temperature, black line). A change in the underlying rate of increase in damage production leads to a change in the slope of the hazard, as observed by Mair et al (insets).

In a classic paper, Mair et al. (*40*) measured the effect of two lifespan-extending interventions in *Drosophila*, lifespan-extending diets (LE) and temperature change, when applied at mid-adulthood (*40*, *41*). They found that the interventions had different effects on lifespan: (i) LE led to rapid switches in mortality rate, and (ii) changing temperature affected the slope of the mortality rate.

These results can be explained by the SR model with rapid turnover (Supplementary Figure 9). A relatively rapid turnover for *Drosophila* means turnover on the order of minutes to hours. We therefore set $\beta = 1\ hr^{-1}$, $\kappa = 1[AU]$, and $\epsilon = 1\ [AU]^2 hr^{-1}$. To fit the survival curve for fully fed flies obtained by Mair et al., we set $\eta = 0.03[AU]hr^{-1}day^{-1}$, and death when $X > X_C$ with $X_C = 15[AU]$. Flies undergoing LE are fit by a lower value, $\eta = 0.02[AU]hr^{-1}day^{-1}$. Note that the purpose here is

to demonstrate that the SR model can capture the behavior of the data, and not to provide accurate estimates for the parameters (the data is insufficient to pin down the parameters).

The LE dietary intervention can be explained by assuming that it changes any of the model parameters. For example, LE can change η in a reversible manner (Supplementary Figure 9A), and hence affect the rate of damage production p. In this case, changing diet leads to damage production $p(t) = \eta_1 t,$ where η_1 is the rate of increase in damage production of the current diet. The rapid turnover of damage rapidly reverts the mortality rates when diet changes (Supplementary Figure 9A). More generally, LE may change any parameter of the SR model, including removal rate β, as long as the effect on the parameter is reversible.

On the other hand, the temperature intervention can be explained by assuming that it affects an underlying damage accumulation rate that sets η (Supplementary Figure 9B), that is, temperature multiplies $\frac{dp}{dt}$. Changing temperature at age *t*' therefore leads to damage production $p(t) = \eta_0 t' + \eta_1(t - t')$, where η_0 was the previous rate of increase in damage production and η_1 the rate after temperature change. This intervention affects the slope of increase in mortality rate with age, but does not revert the mortality rates (Supplementary Figure 9B).

Supplementary Note 9. SR model and temporal scaling of *C. elegans* survival curves

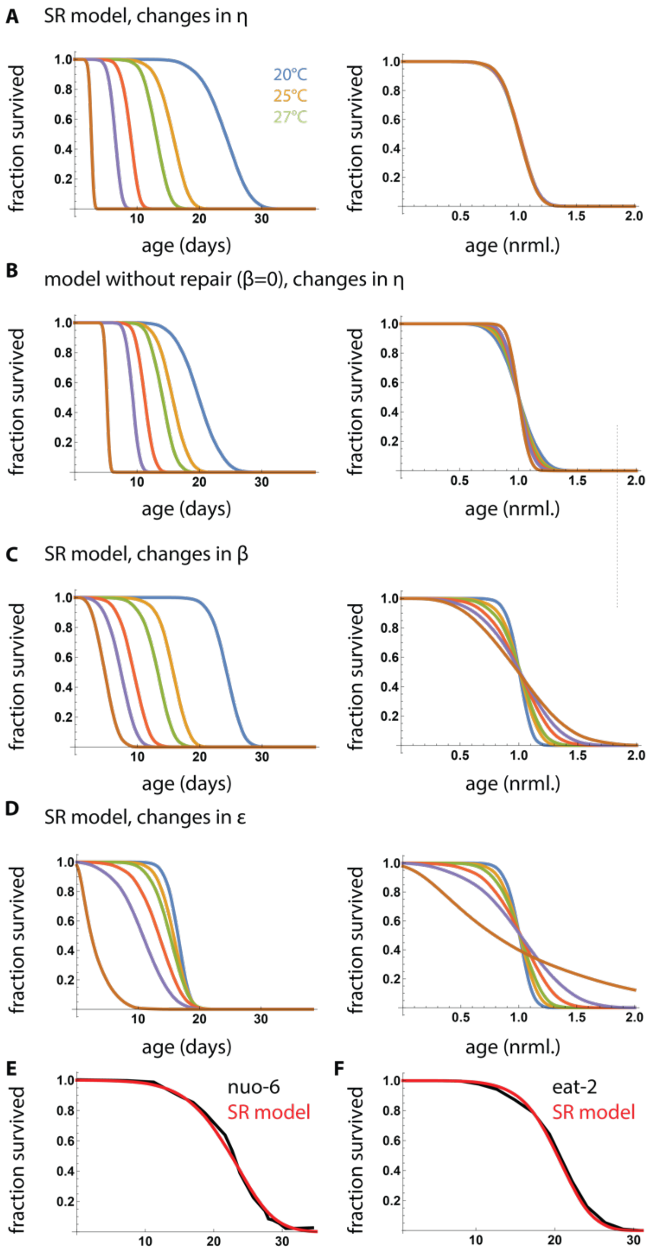

Supplementary Figure 10. SR model with rapid turnover can explain the temporal scaling of survival curves in *C. elegans*. (A) The SR model with saturation and rapid turnover of damage ($\beta = 1\ hr^{-1}$, $\kappa = 1[AU]$, $\epsilon = 1\ [AU]^2hr^{-1}$,$X_C = 20[AU]$), and a slow increase in damage production ($\eta = 0.07[AU]hr^{-1}day^{-1}$) can explain the survival distribution of *C. elegan*s raised at room temperature (25C). Changes in damage production rate η, by multiplying it by a factor λ ($\lambda \in \{0.6, 1, 1.25, 2, 3, 10\}$), lead to lifespan distributions that are temporally scaled, despite a variation of over an order of magnitude in mean lifespan (right panel is age normalized by mean lifespan). (B) A model without damage turnover that was fit to the same survival curve ($\beta = 0,\ \ \eta = 0.006[AU]hr^{-1}day^{-1}, \epsilon = 0.037\ [AU]^2hr^{-1}$) does not show time scaling of survival curves. Time scaling is also not observed for the SR model for changes in β (C) or ϵ (D). Each line represents the simulation of 1000 individuals, time scaling was confirmed according to the statistical criteria of Stroustrup et al. (*42*). (EF) Simultaneous changes in η and in ϵ^{-1} can explain the survival distributions of mutants that do not show temporal scaling – multiplication of η and ϵ^{-1} by 1.9 explains the survival distribution of the nuo-6 mutant (E), while multiplication by 2/3 explains the survival distribution of the eat-2 mutant (F).

In an elegant study, Stroustrup et al. measured lifespan distributions under various conditions that affect mean lifespan, such as temperature, nutrition and mutations (*42*). The aging distributions were different in each condition, but for most perturbations, the curves collapsed to the same curve when normalizing time by a scaling constant. Stroustrup et al. also found that transient temperature interventions shift the lifespan distribution, and concluded that risk of death is determined by a single stochastic process, with a single effective rate constant which is altered by the interventions.

We tested whether the SR model is consistent with these findings, and if so, which parameter is likely to be affected by the lifespan-changing conditions. Note that in this case the form of the damage (the meaning of X in the model) is unknown.

We first estimated a set of physiologically reasonable parameters for the SR model for *C. elegans*. Since *C. elegans* grown at room temperature lives for ~2 weeks on average, a relatively rapid turnover means turnover on the order of minutes to hours. We therefore set $\beta = 1\ hr^{-1}$, $\kappa = 1[AU]$, and $\epsilon = 1\ [AU]^2 hr^{-1}$. To fit the survival curve obtained by Stroustrup et al., we set $\eta = 0.07[AU]hr^{-1}day^{-1}$ and death when $X > X_C$ with $X_C = 20[AU]$.

We find that changes in the parameter η of the SR model can explain the scaling observations of Stroustrup et al. We find that changes in η (i.e. multiplying η by λ, λ∈{0.6,1,1.25,2,3,10}) result in lifespan distributions that are different, but collapse nearly perfectly onto a single distribution when time is normalized by the mean lifespan (Supplementary Figure 10A).

Importantly, time scaling requires rapid turnover. For example, a model without rapid turnover ($\beta = 0$) does not show time scaling of survival curves (Supplementary Figure 10B). Similarly, scaling breaks down when turnover time is comparable to lifespan.

We find that changes in parameters other than η do not provide time scaling of survival curves (Supplementary Figure 10CD). We therefore conclude that the SR model can explain time scaling of survival curves, and identify η as the parameter that is modulated by the interventions.

Generally, the scaling of η needed to fit survival curves in different interventions is inversely proportional to the mean lifespans. For example, for the mutation interventions specified by Stroustrup et al., we find a scaling of $\lambda = 0.75$ for *daf-2(e1368),* $\lambda = 2$ for *daf-16(mu86)* and $\lambda = 1.2$ for *hsf-1(sy441)*. The effects of other time-scaling interventions on η can be similarly calibrated from Supplementary Table 2 in Stroustrup et al. (*42*).

Stroustrup et al. also reported interventions that do not show time scaling. These perturbations may be explained by changes that affect additional parameters besides η. For example, survival of the the*nuo-6 (qm200)* mutant is well fit by multiplying η by $\lambda = \frac{1}{1.9}$ and multiplying noise ϵ by 1.9 (Supplementary Figure 10E), and survival of *eat-2 (ad1116)* mutant is well fit by multiplying η by $\lambda = \frac{2}{3}$ and multiplying noise ϵ by $\frac{3}{2}$ (Supplementary Figure 10F). These findings suggest that it would be intriguing to further explore experimentally how these interventions affect stochastic damage dynamics.

Supplementary References

1. F. d'Adda di Fagagna, Living on a break: cellular senescence as a DNA-damage response. *Nat. Rev. Cancer*. **8**, 512–522 (2008).

2. J. C. Acosta, A. Banito, T. Wuestefeld, A. Georgilis, P. Janich, J. P. Morton, D. Athineos, T.-W. Kang, F. Lasitschka, M. Andrulis, G. Pascual, K. J. Morris, S. Khan, H. Jin, G. Dharmalingam, A. P. Snijders, T. Carroll, D. Capper, C. Pritchard, G. J. Inman, T. Longerich, O. J. Sansom, S. A. Benitah, L. Zender, J. Gil, A complex secretory program orchestrated by the inflammasome controls paracrine senescence. *Nat. Cell Biol.* **15**, 978–990 (2013).

3. D. Aw, A. B. Silva, D. B. Palmer, Immunosenescence: emerging challenges for an ageing population. *Immunology*. **120**, 435–446 (2007).

4. S. C. Grubb, C. J. Bult, M. A. Bogue, Mouse Phenome Database. *Nucleic Acids Res.* **42**, D825–D834 (2014).

5. G. Nelson, J. Wordsworth, C. Wang, D. Jurk, C. Lawless, C. Martin-Ruiz, T. von Zglinicki, A senescent cell bystander effect: senescence-induced senescence: Senescence induces senescence. *Aging Cell*. **11**, 345–349 (2012).

6. D. Jurk, C. Wilson, J. F. Passos, F. Oakley, C. Correia-Melo, L. Greaves, G. Saretzki, C. Fox, C. Lawless, R. Anderson, G. Hewitt, S. L. Pender, N. Fullard, G. Nelson, J. Mann, B. van de Sluis, D. A. Mann, T. von Zglinicki, Chronic inflammation induces telomere dysfunction and accelerates ageing in mice. *Nat. Commun.* **5** (2014), doi:10.1038/ncomms5172.

7. M. Xu, T. Pirtskhalava, J. N. Farr, B. M. Weigand, A. K. Palmer, M. M. Weivoda, C. L. Inman, M. B. Ogrodnik, C. M. Hachfeld, D. G. Fraser, J. L. Onken, K. O. Johnson, G. C. Verzosa, L. G. P. Langhi, M. Weigl, N. Giorgadze, N. K. LeBrasseur, J. D. Miller, D. Jurk, R. J. Singh, D. B. Allison, K. Ejima, G. B. Hubbard, Y. Ikeno, H. Cubro, V. D. Garovic, X. Hou, S. J. Weroha, P. D. Robbins, L. J. Niedernhofer, S. Khosla, T. Tchkonia, J. L. Kirkland, Senolytics improve physical function and increase lifespan in old age. *Nat. Med.* (2018).

8. P. F. L. da Silva, M. Ogrodnik, O. Kucheryavenko, J. Glibert, S. Miwa, K. Cameron, A. Ishaq, G. Saretzki, S. Nagaraja-Grellscheid, G. Nelson, T. von Zglinicki, The bystander effect contributes to the accumulation of senescent cells in vivo. *Aging Cell*, e12848 (2018).

9. M. Xu, T. Pirtskhalava, J. N. Farr, B. M. Weigand, A. K. Palmer, M. M. Weivoda, C. L. Inman, M. B. Ogrodnik, C. M. Hachfeld, D. G. Fraser, J. L. Onken, K. O. Johnson, G. C. Verzosa, L. G. P. Langhi, M. Weigl, N. Giorgadze, N. K. LeBrasseur, J. D. Miller, D. Jurk, R. J. Singh, D. B. Allison, K. Ejima, G. B. Hubbard, Y. Ikeno, H. Cubro, V. D. Garovic, X. Hou, S. J. Weroha, P. D. Robbins, L. J. Niedernhofer, S. Khosla, T. Tchkonia, J. L. Kirkland, Senolytics improve physical function and increase lifespan in old age. *Nat. Med.* **24**, 1246–1256 (2018).

10. K. Yamakoshi, A. Takahashi, F. Hirota, R. Nakayama, N. Ishimaru, Y. Kubo, D. J. Mann, M. Ohmura, A. Hirao, H. Saya, Real-time in vivo imaging of p16Ink4a reveals cross talk with p53. *J Cell Biol*. **186**, 393–407 (2009).

11. C. E. Burd, J. A. Sorrentino, K. S. Clark, D. B. Darr, J. Krishnamurthy, A. M. Deal, N. Bardeesy, D. H. Castrillon, D. H. Beach, N. E. Sharpless, Monitoring Tumorigenesis and Senescence In Vivo with a p16INK4a-Luciferase Model. *Cell*. **152**, 340–351 (2013).

12. N. Berglund, Kramers' law: Validity, derivations and generalisations. *ArXiv11065799 Math-Ph* (2011) (available at http://arxiv.org/abs/1106.5799).

13. H. A. Kramers, Brownian motion in a field of force and the diffusion model of chemical reactions. *Physica*. **7**, 284–304 (1940).

14. B. Gompertz, On the Nature of the Function Expressive of the Law of Human Mortality, and on a New Mode of Determining the Value of Life Contingencies. *Philos. Trans. R. Soc. Lond.* **115**, 513–583 (1825).

15. T. B. L. Kirkwood, Deciphering death: a commentary on Gompertz (1825) 'On the nature of the function expressive of the law of human mortality, and on a new mode of determining the value of life contingencies.' *Phil Trans R Soc B*. **370**, 20140379 (2015).

16. T. I. Missov, A. Lenart, Gompertz–Makeham life expectancies: Expressions and applications. *Theor. Popul. Biol.* **90**, 29–35 (2013).

17. S. J. Olshansky, B. A. Carnes, Ever since gompertz. *Demography*. **34**, 1–15 (1997).

18. A. A. Sas, H. Snieder, J. Korf, Gompertz' survivorship law as an intrinsic principle of aging. *Med. Hypotheses*. **78**, 659–663 (2012).

19. J. S. Weitz, H. B. Fraser, Explaining mortality rate plateaus. *Proc. Natl. Acad. Sci.* **98**, 15383–15386 (2001).

20. E. Barbi, F. Lagona, M. Marsili, J. W. Vaupel, K. W. Wachter, The plateau of human mortality: Demography of longevity pioneers. *Science*. **360**, 1459–1461 (2018).

21. J. L. Folks, R. S. Chhikara, The Inverse Gaussian Distribution and Its Statistical Application--A Review. *J. R. Stat. Soc. Ser. B Methodol.* **40**, 263–289 (1978).

22. A. Biran, L. Zada, P. Abou Karam, E. Vadai, L. Roitman, Y. Ovadya, Z. Porat, V. Krizhanovsky, Quantitative identification of senescent cells in aging and disease. *Aging Cell*. **16**, 661–671 (2017).

23. M. P. Baar, R. M. C. Brandt, D. A. Putavet, J. D. D. Klein, K. W. J. Derks, B. R. M. Bourgeois, S. Stryeck, Y. Rijksen, H. van Willigenburg, D. A. Feijtel, I. van der Pluijm, J. Essers, W. A. van Cappellen, W. F. van IJcken, A. B. Houtsmuller, J. Pothof, R. W. F. de Bruin, T. Madl, J. H. J. Hoeijmakers, J. Campisi, P. L. J. de Keizer, Targeted Apoptosis of Senescent Cells Restores Tissue Homeostasis in Response to Chemotoxicity and Aging. *Cell*. **169**, 132-147.e16 (2017).

24. J. Chang, Y. Wang, L. Shao, R.-M. Laberge, M. Demaria, J. Campisi, K. Janakiraman, N. E. Sharpless, S. Ding, W. Feng, Y. Luo, X. Wang, N. Aykin-Burns, K. Krager, U. Ponnappan, M. Hauer-Jensen, A. Meng, D. Zhou, Clearance of senescent cells by ABT263 rejuvenates aged hematopoietic stem cells in mice. *Nat. Med.* **22**, 78–83 (2016).

25. J. L. Kirkland, T. Tchkonia, Cellular Senescence: A Translational Perspective. *EBioMedicine*. **21**, 21–28 (2017).

26. J. L. Kirkland, T. Tchkonia, Y. Zhu, L. J. Niedernhofer, P. D. Robbins, The Clinical Potential of Senolytic Drugs. *J. Am. Geriatr. Soc.* **65**, 2297–2301 (2017).

27. C. M. Roos, B. Zhang, A. K. Palmer, M. B. Ogrodnik, T. Pirtskhalava, N. M. Thalji, M. Hagler, D. Jurk, L. A. Smith, G. Casaclang-Verzosa, Y. Zhu, M. J. Schafer, T. Tchkonia, J. L. Kirkland, J. D. Miller, Chronic senolytic treatment alleviates established vasomotor dysfunction in aged or atherosclerotic mice. *Aging Cell*. **15**, 973–977 (2016).

28. Y. Zhu, T. Tchkonia, T. Pirtskhalava, A. C. Gower, H. Ding, N. Giorgadze, A. K. Palmer, Y. Ikeno, G. B. Hubbard, M. Lenburg, S. P. O'Hara, N. F. LaRusso, J. D. Miller, C. M. Roos, G. C. Verzosa, N. K. LeBrasseur, J. D. Wren, J. N. Farr, S. Khosla, M. B. Stout, S. J. McGowan, H. Fuhrmann-Stroissnigg, A. U. Gurkar, J. Zhao, D. Colangelo, A. Dorronsoro, Y. Y. Ling, A. S. Barghouthy, D. C. Navarro, T. Sano, P. D. Robbins, L. J. Niedernhofer, J. L. Kirkland, The Achilles' heel of senescent cells: from transcriptome to senolytic drugs. *Aging Cell*. **14**, 644–658 (2015).

29. Y. Zhu, T. Tchkonia, H. Fuhrmann-Stroissnigg, H. M. Dai, Y. Y. Ling, M. B. Stout, T. Pirtskhalava, N. Giorgadze, K. O. Johnson, C. B. Giles, J. D. Wren, L. J. Niedernhofer, P. D. Robbins, J. L. Kirkland, Identification of a novel senolytic agent, navitoclax, targeting the Bcl-2 family of anti-apoptotic factors. *Aging Cell*. **15**, 428–435 (2016).

30. D. J. Baker, B. G. Childs, M. Durik, M. E. Wijers, C. J. Sieben, J. Zhong, R. A. Saltness, K. B. Jeganathan, G. C. Verzosa, A. Pezeshki, K. Khazaie, J. D. Miller, J. M. van Deursen, Naturally occurring p16(Ink4a)-positive cells shorten healthy lifespan. *Nature*. **530**, 184–189 (2016).

31. D. A. Wise, Ed., *Studies in the economics of aging* (University of Chicago Press, Chicago, 1994), *A National Bureau of Economic Research project report*.

32. J. R. Wilmoth, L. J. Deegan, H. Lundström, S. Horiuchi, Increase of maximum life-span in Sweden, 1861-1999. *Science*. **289**, 2366–2368 (2000).

33. A. I. Yashin, A. S. Begun, S. I. Boiko, S. V. Ukraintseva, J. Oeppen, New age patterns of survival improvement in Sweden: do they characterize changes in individual aging? *Mech. Ageing Dev.* **123**, 637–647 (2002).

34. W. M. Makeham, On the Law of Mortality and Construction of Annuity Tables. *Assur. Mag. J. Inst. Actuar.* **8**, 301–310 (1860).

35. Y. Liu, H. K. Sanoff, H. Cho, C. E. Burd, C. Torrice, J. G. Ibrahim, N. E. Thomas, N. E. Sharpless, Expression of p16INK4a in peripheral blood T-cells is a biomarker of human aging. *Aging Cell*. **8**, 439–448 (2009).

36. S. Zhou, J. S. Greenberger, M. W. Epperly, J. P. Goff, C. Adler, M. S. Leboff, J. Glowacki, Age-related intrinsic changes in human bone-marrow-derived mesenchymal stem cells and their differentiation to osteoblasts. *Aging Cell*. **7**, 335–343 (2008).

37. A. Melk, B. M. W. Schmidt, O. Takeuchi, B. Sawitzki, D. C. Rayner, P. F. Halloran, Expression of p16INK4a and other cell cycle regulator and senescence associated genes in aging human kidney. *Kidney Int.* **65**, 510–520 (2004).

38. Z.-Y. Li, Z.-L. Chen, T. Zhang, C. Wei, W.-Y. Shi, TGF-β and NF-κB signaling pathway crosstalk potentiates corneal epithelial senescence through an RNA stress response. *Aging*. **8**, 2337–2354 (2016).

39. J. A. Martin, J. A. Buckwalter, Telomere erosion and senescence in human articular cartilage chondrocytes. *J. Gerontol. A. Biol. Sci. Med. Sci.* **56**, B172-179 (2001).

40. W. Mair, Demography of Dietary Restriction and Death in Drosophila. *Science*. **301**, 1731–1733 (2003).

41. R. C. Grandison, M. D. W. Piper, L. Partridge, Amino acid imbalance explains extension of lifespan by dietary restriction in Drosophila. *Nature*. **462**, 1061–1064 (2009).

42. N. Stroustrup, W. E. Anthony, Z. M. Nash, V. Gowda, A. Gomez, I. F. López-Moyado, J. Apfeld, W. Fontana, The temporal scaling of Caenorhabditis elegans ageing. *Nature*. **530**, 103–107 (2016).
