# Supplementary material for: Senescent cell turnover slows with age providing an explanation for the Gompertz law
Source: Nat Commun. 2019 Dec 2;10:5495. doi: 10.1038/s41467-019-13192-4 (PMC6889273; doi:10.1038/s41467-019-13192-4)
Supplement: Supplementary file 3 — Reporting Summary [file 41467_2019_13192_MOESM3_ESM.pdf]

nature research

Corresponding author(s): Uri Alon

Last updated by author(s): 8/10/2019

Reporting Summary

Nature Research wishes to improve the reproducibility of the work that we publish. This form provides structure for consistency and transparency in reporting. For further information on Nature Research policies, see Authors & Referees and the Editorial Policy Checklist.

Statistics

For all statistical analyses, confirm that the following items are present in the figure legend, table legend, main text, or Methods section.

n/a	Confirmed	
☐	☒	The exact sample size (n) for each experimental group/condition, given as a discrete number and unit of measurement
☐	☒	A statement on whether measurements were taken from distinct samples or whether the same sample was measured repeatedly
☐	☒	The statistical test(s) used AND whether they are one- or two-sided<br>*Only common tests should be described solely by name; describe more complex techniques in the Methods section.*
☐	☒	A description of all covariates tested
☐	☒	A description of any assumptions or corrections, such as tests of normality and adjustment for multiple comparisons
☐	☒	A full description of the statistical parameters including central tendency (e.g. means) or other basic estimates (e.g. regression coefficient) AND variation (e.g. standard deviation) or associated estimates of uncertainty (e.g. confidence intervals)
☐	☒	For null hypothesis testing, the test statistic (e.g. F, t, r) with confidence intervals, effect sizes, degrees of freedom and P value noted<br>*Give P values as exact values whenever suitable.*
☐	☒	For Bayesian analysis, information on the choice of priors and Markov chain Monte Carlo settings
☐	☒	For hierarchical and complex designs, identification of the appropriate level for tests and full reporting of outcomes
☐	☒	Estimates of effect sizes (e.g. Cohen's d, Pearson's r), indicating how they were calculated

Our web collection on statistics for biologists contains articles on many of the points above.

Software and code

Policy information about availability of computer code

Data collection	No open-source or custom code was used to collect data for this paper.
Data analysis	Data analysis was conducted using Mathematica version 11.3 and IDEAS 6.2. Custom code was written in Mathematica 11.3 (Wolfram) and can be found in the following link—<br>https://github.com/omerka-weizmann/sncdynamics<br>The analysis file contains functions for simulating circuit topologies, obtaining mortality statistics, computing log-likelihood of stochastic trajectories, and computing various summary statistics of stochastic trajectory data.

For manuscripts utilizing custom algorithms or software that are central to the research but not yet described in published literature, software must be made available to editors/reviewers. We strongly encourage code deposition in a community repository (e.g. GitHub). See the Nature Research guidelines for submitting code & software for further information.

Data

Policy information about availability of data

All manuscripts must include a data availability statement. This statement should provide the following information, where applicable:

- Accession codes, unique identifiers, or web links for publicly available datasets
- A list of figures that have associated raw data
- A description of any restrictions on data availability

The source data underlying Figure 2ACDEFG, Figure 3C, Figure 4BCD, and Supplementary Figure 2A are available as a Source File. All other data are available from the corresponding author upon request.

Field-specific reporting

Please select the one below that is the best fit for your research. If you are not sure, read the appropriate sections before making your selection.

☒ Life sciences ☐ Behavioural & social sciences ☐ Ecological, evolutionary & environmental sciences

For a reference copy of the document with all sections, see nature.com/documents/nr-reporting-summary-flat.pdf

Life sciences study design

All studies must disclose on these points even when the disclosure is negative.

Sample size	The mice analyzed were 3-month-olds treated with 1.5U/kg (n=17), 3-month-olds treated with PBS (n=13), 22-month-olds treated with 1.5U/kg (n=13), and 22-month-olds treated with PBS (n=6).
Data exclusions	We did not exclude any data points from the analysis.
Replication	Experiments were performed with replications. The replications were successful.
Randomization	Experiments involved two treatment groups: Bleomycin and PBS. The mice were randomly divided into two groups before the treatment. Outliers based upon weight were excluded from the study.
Blinding	Not relevant

Reporting for specific materials, systems and methods

We require information from authors about some types of materials, experimental systems and methods used in many studies. Here, indicate whether each material, system or method listed is relevant to your study. If you are not sure if a list item applies to your research, read the appropriate section before selecting a response.

Materials & experimental systems

n/a	Involved in the study	
☐	☒	Antibodies
☒	☐	Eukaryotic cell lines
☒	☐	Palaeontology
☐	☒	Animals and other organisms
☒	☐	Human research participants
☒	☐	Clinical data

Methods

n/a	Involved in the study	
☒	☐	ChIP-seq
☒	☐	Flow cytometry
☒	☐	MRI-based neuroimaging

Antibodies

Antibodies used	The following antibodies were used in the experiment:<br>1. PE-conjugated pan-cytokeratin (ab52460, Abcam)<br>2. HMGB1 (ab18256, Abcam)<br>3. Qdot605 labeled Goat Anti-Rabbit antibody (Q11402MP, ThermoFisher)
Validation	B. Anat et al., Quantitative identification of senescent cells in aging and disease. Aging cell. 16.4, 661-671 (2017).

Animals and other organisms

Policy information about studies involving animals; ARRIVE guidelines recommended for reporting animal research

Laboratory animals	3-month-old (young) and 22-month-old (aged) C57BL6 mice were used in the experiments.
Wild animals	The study did not involve wild animals.
Field-collected samples	The study did not involve samples collected from the field.
Ethics oversight	The experiments were approved by Weizmann Institute's Institutional Animal Care and Use Committee.

Note that full information on the approval of the study protocol must also be provided in the manuscript.
